# ^18^F-PSMA-1007 PET/CT-derived semi-quantitative parameters for risk stratification of newly diagnosed prostate cancer

**DOI:** 10.3389/fonc.2022.1025930

**Published:** 2022-12-07

**Authors:** Siying Dong, Yanmei Li, Jian Chen, Yongliang Li, Pengfei Yang, Juan Li

**Affiliations:** ^1^ Department of Nuclear Medicine, General Hospital of Ningxia Medical University, Yinchuan, China; ^2^ College of Clinical Medicine, Ningxia Medical University, Yinchuan, China

**Keywords:** ^18^F-PSMA-1007, PET/CT, prostate cancer, risk stratification, semi-quantitative parameters

## Abstract

**Purpose:**

This study aimed to assess the value of ^18^F-PSMA-1007 positron emission tomography/computed tomography (PET/CT)-derived semi-quantitative parameters of primary tumor for risk stratification of newly diagnosed prostate cancer (PCa).

**Methods:**

Sixty patients referred for ^18^F-PSMA-1007 PET/CT imaging for primary PCa were retrospectively analyzed and classified into the low-intermediate-risk (LIR) or high-risk (HR) group. The maximum standardized uptake value (SUVmax) of primary tumor, prostate total lesion PSMA (TL-PSMAp), and prostate PSMA-tumor volume (PSMA-TVp) were measured, and group differences were evaluated using the Mann–Whitney U test. Spearman’s correlation was performed to assess the correlation between the above parameters with prostate-specific antigen (PSA) levels and Gleason score (GS). Receiver operating characteristic (ROC) curve analysis was used to determine optimal cut-off values for SUVmax, TL-PSMAp, and PSMA-TVp to identify high-risk PCa and compare diagnostic efficacy.

**Results:**

Among 60 patients, 46 were assigned to the HR group and 16 to the LIR group. In all patients, SUVmax, TL-PSMAp, and PSMA-TVp were moderately correlated with pre-treatment PSA values (*r* = 0.411, *p* = 0.001; *r* = 0.663, *p* < 0.001; and *r* = 0.549, *p* < 0.001, respectively). SUVmax and TL-PSMAp were moderately correlated with GS (*r* = 0.457 and *r* = 0.448, respectively; *p* < 0.001), while PSMA-TVp was weakly correlated with GS (*r* = 0.285, *p* = 0.027). In the ROC curve analysis, the optimal cut-off values of SUVmax, TL-PSMAp, and PSMA-TVp for identifying high-risk PCa were 9.61, 59.62, and 10.27, respectively, and the areas under the operating curve were 0.828, 0.901, and 0.809, respectively. The sensitivities of SUVmax, TL-PSMAp, and PSMA-TVp were 91.03%, 71.74%, and 63.04%, respectively, and the specificities were 71.43%, 100.00%, and 92.86%, respectively.

**Conclusions:**

TL-PSMAp had a superior ability to identify high-risk PCa. The semi-quantitative parameters of primary tumor on ^18^F-PSMA-1007 PET/CT imaging can be an objective imaging reference index to determine PCa risk stratification.

## Introduction

According to the International Agency for Research on Cancer’s GLOBOCAN 2020 data, prostate cancer (PCa) is the most common cancer in males, with the fourth highest morbidity rate and eighth highest mortality rate of all cancers worldwide (1). The biological behavior of PCa with different degrees of malignancy varies greatly and directly affects treatment decisions and prognoses. For patients with low- to intermediate-risk PCa, active monitoring, radical surgery, and radical radiotherapy can provide a better prognosis. High-risk PCa is associated with an increased risk of metastasis, recurrence, and death (2). Thus, early identification of high-risk PCa is crucial to improving patient prognosis and survival. Currently, the D’Amico risk classification, EAU-EanM-Estro-Esur-SIOG Guidelines on Prostate Cancer, and National Comprehensive Cancer Network (NCCN) Guidelines for Prostate Cancer are commonly used for risk classification, and the main parameters evaluated include pretreatment prostate-specific antigen (PSA) level, Gleason score (GS), and clinical T stage (3–5). However, there is no agreement yet upon the above guidelines on the minimum requirements for the clinical T stage of high-risk PCa, with the first two T stages ≥2c and the latter T stage ≥3a. The accuracy of T staging based on subjective clinical judgments is also not always reliable. Therefore, objective and accurate non-invasive imaging indicators are urgently needed as reference standards for the diagnosis of high-risk PCa.

Prostate-specific membrane antigen (PSMA), also known as folate hydrolase I or glutamate carboxypeptidase II, plays a vital role in PCa imaging and treatment. PSMA is highly expressed in PCa cells, whereas levels are low in benign prostatic tissues (6). Several studies have shown increased PSMA expression in advanced-stage, poorly differentiated, and castration-resistant PCa (7). In patients with advanced metastatic disease prior to PSMA radioligand treatment, high PSMA uptake appeared to predict adverse outcomes (8, 9). PSMA positron emission tomography/computed tomography (PET/CT) has been applied to a variety of PCa assessment and management strategies, such as primary staging, lymph nodal and distant staging, treatment planning, treatment response evaluation, and localization of biochemical recurrence (7, 10–16).

Another benefit of PET imaging is that imaging parameters can be quantified by calculating standardized uptake value (SUV) and volume parameters, such as PSMA-tumor volume (PSMA-TV), and total lesion PSMA (TL-PSMA). SUV is the most commonly used semi-quantitative parameter to measure the uptake of radioactive tracers (17), which shows the ratio of the activity concentration in the region of interest to the average activity concentration in the whole body. The maximum SUV (SUVmax), a commonly used convenience parameter in clinical practice, is the highest value of the metabolic activity of tumor tissue and is often used to identify benign and malignant prostate lesions (18, 19) and prostate cancer grade (20). Some studies have shown that PSMA-PET/CT SUVmax has a higher sensitivity and can be an “imaging biomarker” for primary PCa risk stratification (18, 21, 22). PSMA-TV represents the hypermetabolic tumor volume, which reflects the number of hypermetabolic cells and can indirectly reflect the size of tumor burden. TL-PSMA is defined as the product of the mean SUV and the MTV, representing the sum of the SUV within the lesion. As volume parameters are considered to be more comprehensive and reflective of metabolic tumor burden than SUVmax (17), clinicians can obtain more detailed tumor information for clinical management. Bettermann et al. (23) found that PSMA PET was more accurate than mpMRI in delineating intraprostatic gross tumor volume (GTV), especially with a higher sensitivity. Ferraro et al. (24) demonstrated that PSMA PET-derived Semi-quantitative parameters could help to reduce unnecessary extended pelvic lymph node dissection in patients with intermediate-or high-risk prostate cancer. Yildirim et al. (25) found that PSMA PET volume parameters could be used to distinguish patients with rapid recurrence from others. Some authors showed that whole-body PSMA-TV and TL-PSMA values are being used for treatment response evaluation in patients with metastatic PCa and are among the most valuable parameters for evaluation of therapy response and prediction of survival rates (26, 27). Liu et al. (28) indicated that PSMA-TV and TL-PSMA can effectively predict high-risk PCa and metastasis risk, while SUVmax can only predict high-risk PCa.

Recently, a novel PSMA-based radiopharmaceutical, ^18^F-PSMA-1007, has emerged, with several irreplaceable advantages over ^68^Ga-PSMA-11-617. For example, ^18^F-PSMA-1007 is more accessible, with a higher yield, longer half-life, and higher physical spatial resolution (29). Another clear advantage of ^18^F-PSMA-1007 is the absence of renal excretion and low urinary activity, which can be advantageous for the detection and diagnosis of lesions near the ureter or bladder (30).

Until now, few studies focusing on the newly developed radiotracer ^18^F-PSMA-1007 have been reported, and the value of the volume parameters PSMA-TV and TL-PSMA for risk stratification was not discussed. Therefore, this study aimed to analyze the correlation between ^18^F-PSMA-1007 PET/CT-derived SUVmax, TL-PSMAp, and PSMA-TVp with serum PSA values in PCa patients prior to treatment and Gleason scores and to assess the value of this imaging method for PCa risk stratification.

## Materials and methods

### Study population

This retrospective study population comprised 60 men referred for ^18^F-PSMA-1007 PET/CT imaging of primary PCa between May 2020 and April 2022. The inclusion criteria were as follows: (1) confirmation of PCa by needle biopsy of the prostate and (2) complete clinical data were available for all patients. The exclusion criteria were as follows: (1) >4 weeks between PSA values and ^18^F-PSMA-1007 PET/CT imaging, (2) patients referred for treatment prior to study enrolment, and (3) patients with a previous history of other cancers. According to the NCCN Guidelines for Prostate Cancer (5), all patients were divided into low-intermediate-risk (LIR) or high-risk (HR) groups. Patients in the LIR group were required to meet the following criteria: (1) PSA ≤20 ng/ml; (2) GS <8; and (3) clinical stage cT1-cT2c. Similarly, patients in the HR group were required to meet at least one of the following criteria: (1) PSA >20 ng/ml, (2) GS 8–10, or (3) clinical stage ≥cT3a. This study was approved by the Ethics Committee of the General Hospital of Ningxia Medical University (approval number: 2020-083). This study was performed in line with the principles of the Declaration of Helsinki the ethical standards of the institutional and/or national research committees. All patients provided informed written consent.

### Image acquisition

^18^F was produced by a Sumitomo HM-10 cyclotron (Sumitomo Heavy Industries, Tokyo, Japan). The reagent kit and PSMA-1007 precursor were obtained from ABX Advanced Biochemical Compounds (Radeberg, Germany). ^18^F-PSMA-1007 was synthesized using PET-IFB-X5 automatic synthesis (Shaanxi Zhengze Biotechnology Co., Ltd., Xi’an, China). ^18^F-PSMA-1007 radiochemical purity was >95%, measured by high-performance liquid chromatography.

PET/CT images were acquired from a Discovery VCT PET/CT scanner (GE HealthCare, Chicago, IL, USA). The scans were performed for a median time of 118 minutes (range: 71–178 minutes) after an injection of 4.81 MBq/kg ^18^F-PSMA-1007 (median activity: 336.6 MBq; range: 229.4.0–451.4 MBq). Patients were placed in the supine position and scanned from the base of the skull to the proximal femora (7–8 bed positions; 2.5 min per bed position; 3D acquisition mode; 700 mm field of view; 128 × 128 matrix; 3.27 mm slice thickness and interval). Attenuation correction of the PET images was carried out using CT data. Attenuation correction was based on non-enhanced low-dose CT (120 kV tube voltage; 30–210 mA automatic tube current modulation; 0.516:1 pitch; 0.5 s tube rotation speed; 3.75 mm section thickness; 3.75 mm reconstruction thickness). PET images were reconstructed using the ordered subset expectation maximization reconstruction algorithm (2 iterations; 28 subsets) at a MedEx workstation (MedEx (Beijing) Technology Limited Corporation, Beijing, China).

### Image analysis

All ^18^F-PSMA-PET/CT images were analyzed with the MedEx workstation, which allowed for the review of PET, CT, and fused imaging data in axial, coronal, and sagittal slices. PET images were interpreted independently by two experienced nuclear medicine physicians with over five years of clinical experience who were blinded to all relevant clinical statistics. Any disagreements were resolved by consensus. Any focal tracer uptake higher than the surrounding prostate tissue or background and not associated with physiological uptake or known pitfalls was considered suspicious for malignancy (31).

**Figure 1:**
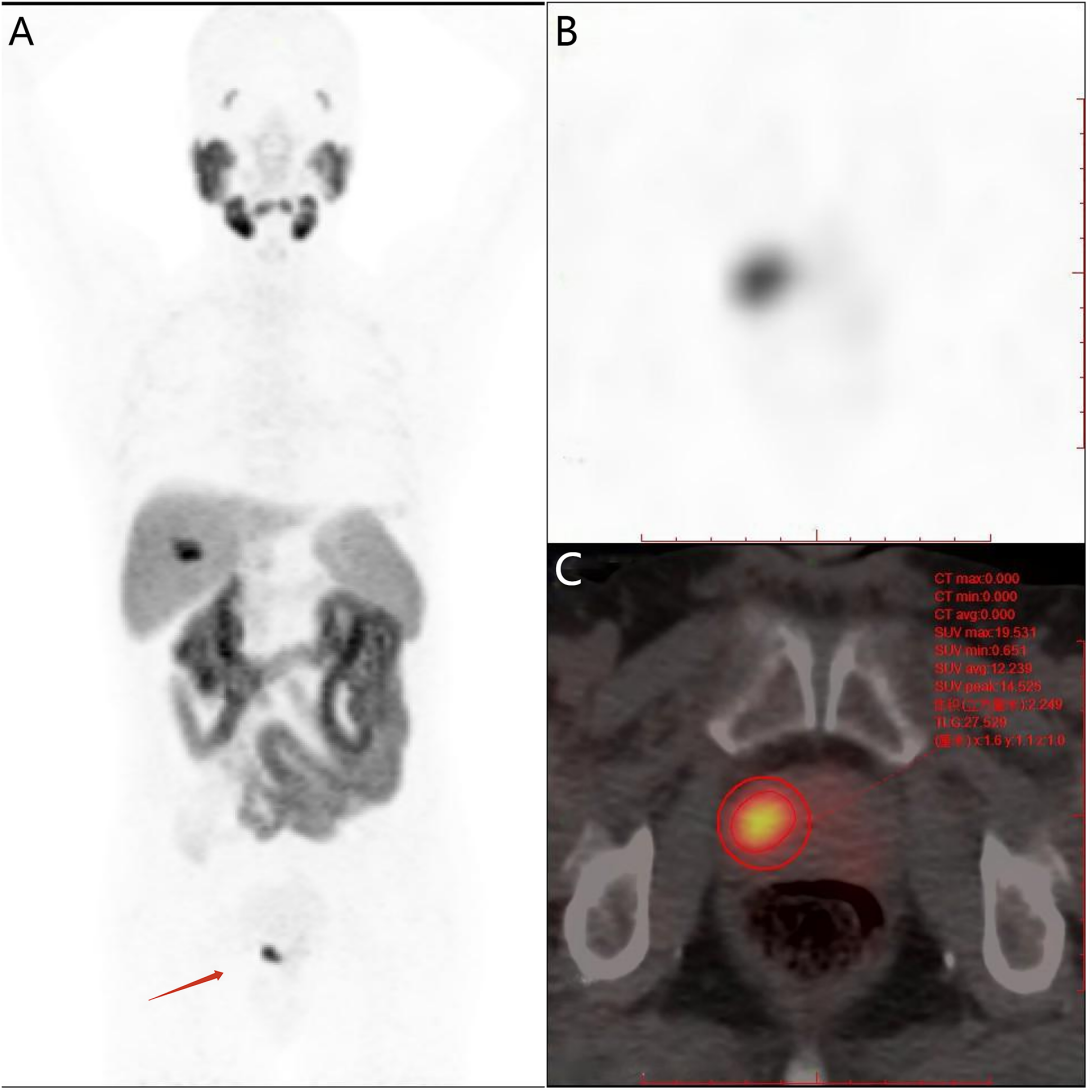
Semi-quantitative parameters of the primary prostate tumor were measured on ^18^F-PSMA-1007 PET/CT imaging by the 3D sketching method. **(A)** Primary prostate cancer was revealed on whole-body maximum intensity projection (MIP) imaging (red arrow); **(B)** the prostate positive lesion was shown on axial PET imaging; **(C)** the volume of interest of the prostate lesion was obtained on axial fusion image (smaller red circle surrounding the lesion). SUVmax and PSMA-derived tumor volume (PSMA-TV) of the lesion were obtained by the threshold method as 19.25 and 2.39 cm^3^, respectively, and the workstation automatically calculated total lesion PSMA (TL-PSMA) of the lesion as 27.83 cm^3^.

The point of the maximum value of tracer uptake within the prostate positive lesion was selected as the center and the volume of interest (VOI) of the primary prostate tumor was drawn by 3D sketching method, manually excluding the tracer uptake of surrounding normal tissues (bladder and/or rectum) and manually adjusting VOIs to match the edge of the positive lesion (28). With the threshold method (40% SUVmax), the PSMA-derived prostate tumor volume (PSMA-TVp) and SUVmean values were obtained within the VOI of the positive lesion. TL-PSMAp values were calculated by multiplying the SUVmean and PSMA-TVp values (**Figure 1**).

### Statistical analyses

Statistical analyses were performed using SPSS v.26 (IBM Corporation, Armonk, NY, USA) and MedCalc v.20.1 (MedCalc Software, Ostend, Belgium). Statistical significance was set at *p <*0.05. Normally distributed data were presented as mean ± standard deviation, and non-normally distributed data were presented as median (range). Using Spearman’s correlation coefficient for numerical variables, we correlated the clinical and laboratory parameters (PSA level and GS) with SUVmax and volumetric parameters (TL-PSMAp and PSMA-TVp). Differences in metabolic parameters between the LIR and HR groups were evaluated using the Mann–Whitney U test. Receiver operating characteristic (ROC) curves were plotted to determine the optimal cut-off values of the PSMA-PET parameters for the detection of high-risk PCa and compare the diagnostic efficacy of these parameters.

## Results

The clinical characteristics and PSMA-PET parameters of the 60 patients are summarized in **Table 1**. Among the 60 patients, 23.3% (14/60) were assigned to the LIR group and 76.7% (46/60) to the HR group.

**Table 1:** Clinical characteristics and primary tumor semi-quantitative PSMA-PET parameters.

Patients (n)	60
Age (years)	68 (54–87)
PSA (ng/ml)	37.24 (5.29–113.59)
PSA ≤20 (ng/ml)	20
PSA >20 (ng/ml)	40
Primary Gleason Score	
G6	9
G7	17
G8	10
G9	16
G10	8
Clinical T stage	
T2a	8
T2b	5
T2c	12
T3a	4
T3b	16
T4	15
Risk groups	
LIR	14
HR	46
SUVmax	14.53 (3.28 –99.95)
TL-PSMAp (cm^3^)	83.50 (5.52–858.72)
PSMA-TVp (cm^3^)	10.27 (0.98–58.87)

Non-normally distributed data are presented as median (range).

PSMA, prostate-specific membrane antigen; PSA, prostate-specific antigen; LIR, low-intermediate-risk; HR, high-risk; SUVmax, maximum standardized uptake value; TL-PSMAp, prostate total lesion PSMA; PSMA-TVp, prostate PSMA-tumor volume; PET, positron emission tomography.

### Comparison of primary tumor semi-quantitative PSMA-PET parameters in patients in the LIR and HR groups

The median SUVmax value obtained from patients in the HR group was higher than that from patients in the LIR group (16.17 *vs*. 7.73, respectively), and the difference was significant (*p* < 0.001). Furthermore, there were statistically significant differences in median TL-PSMAp and PSMA-TVp values between the HR and LIR groups (114.35 *vs*. 27.74 and 12.52 *vs*. 5.97, respectively; *p* < 0.001; **Table 2**).

**Table 2:** Comparison of primary tumor semi-quantitative PSMA-PET parameters in prostate cancer patients with different risk stratification.

risk stratification	SUVmax	TL-PSMAp (cm^3^)	PSMA-TVp (cm^3^)
LIR (n = 14)	7.73 (3.28–31.05)	27.74 (5.52–56.83)	5.97 (0.98–11.25)
HR (n = 46)	16.17 (4.50–99.95)	114.35 (15.60–858.72)	12.52 (1.47–58.87)
*p-*value	<0.001	<0.001	<0.001

Non-normally distributed data are presented as median (range).

SUVmax, maximum standardized uptake value; PSMA, prostate-specific membrane antigen; TL-PSMAp, prostate total lesion PSMA; PSMA-TVp, prostate PSMA-tumor volume; LIR, low-intermediate-risk; HR, high-risk; PET, positron emission tomography.

### Correlation of serum PSA levels and Gleason scores with primary tumor semi-quantitative PSMA-PET parameters

**Figure 2:**
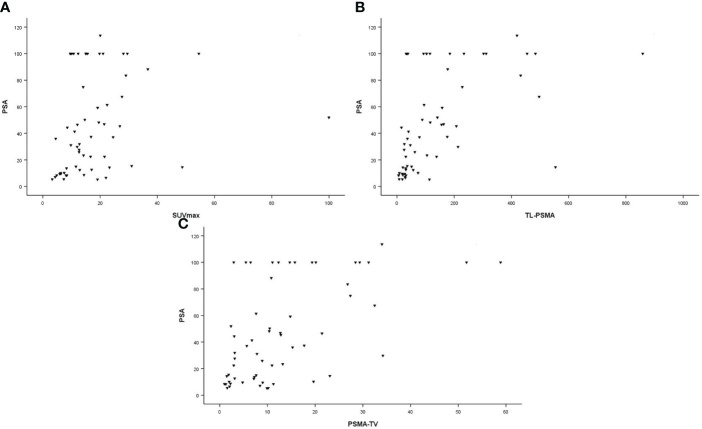
Correlation between PSA values (ng/ml) and primary tumor prostate-specific membrane antigen (PSMA) PET parameters. **(A)** Maximum standardized uptake value (SUVmax; *r* = 0.411; *p* = 0.001). **(B)** Prostate total lesion PSMA (TL-PSMAp [cm^3^]; *r* = 0.663; *p* < 0.001). **(C)** prostate PSMA-tumor volume (PSMA-TVp [cm^3^]; *r* = 0.549; *p* < 0.001).

**Figure 3:**
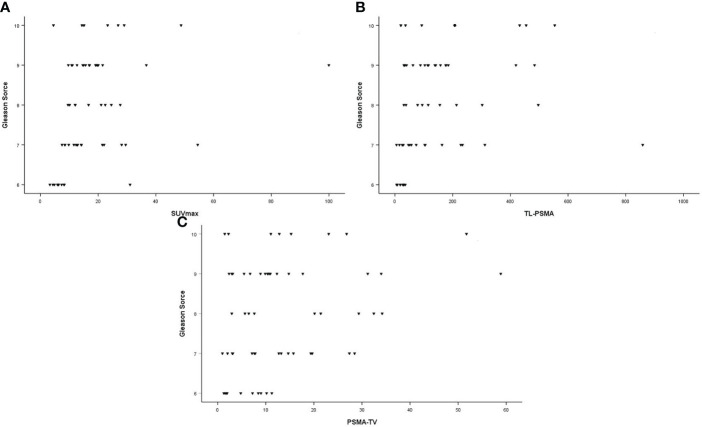
Correlation between Gleason scores and primary tumor prostate-specific membrane antigen (PSMA) PET parameters. **(A)** Maximum standardized uptake value (SUVmax; *r* = 0.457; *p* < 0.001). **(B)** Prostate total lesion PSMA (TL-PSMAp [cm^3^]; *r* = 0.448; *p* < 0.001). **(C)** Prostate PSMA-tumor volume (PSMA-TVp [cm^3^]; *r* =0.285; *p* = 0.027).

In all patients, SUVmax, TL-PSMAp, and PSMA-TVp were moderately correlated with PSA values (*r* = 0.411, *p* = 0.001; *r* = 0.663, *p* < 0.001; and *r* = 0.549, *p* < 0.001, respectively). These correlations are illustrated in **Figure 2**. There was a moderate correlation between GS and SUVmax and TL-PSMAp (*r* = 0.457, *p* < 0.001 and *r* = 0.448, *p* < 0.001, respectively). PSMA-TVp showed a weak correlation with GS (*r* = 0.285, *p* = 0.027) (**Figure 3**).

**Figure 4:**
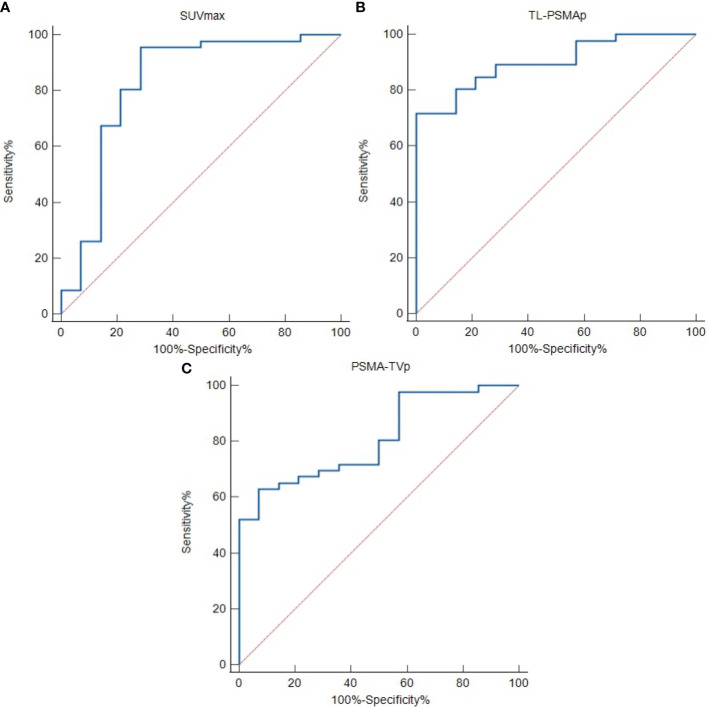
Receiver operating characteristic (ROC) curves to detect high-risk prostate cancer using semi-quantitative prostate-specific membrane antigen (PSMA) PET parameters. **(A)** Maximum standardized uptake value (SUVmax). **(B)** Prostate total lesion PSMA (TL-PSMAp). **(C)** Prostate PSMA-tumor volume (PSMA-TVp).

ROC curve analysis of the differentiation of HR from LIR PCa revealed good diagnostic efficacy, sensitivity, and specificity for all investigated imaging parameters. The optimal cut-off values of SUVmax, TL-PSMAp, and PSMA-TVp were 9.61, 59.62, and 10.27, respectively. The ROC curve plots are depicted in **Figure 4**, and the respective values are shown in **Table 3**.

**Table 3:** AUC characteristics for the investigated semi-quantitative PSMA-PET parameters.

parameter	AUC	Sensitivity	Specificity	95% CI	*p-*value
SUVmax	0.828	91.03%	71.43%	0.673–0.982	<0.001
TL-PSMAp	0.901	71.74%	100.00%	0.824–0.978	<0.001
PSMA-TVp	0.809	63.04%	92.86%	0.695–0.923	<0.001

AUC, area under the curve; CI, confidence interval; SUVmax, maximum standardized uptake value; PSMA, prostate-specific membrane antigen; TL-PSMAp, prostate total lesion PSMA; PSMA-TVp, prostate PSMA-tumor volume; PET, positron emission tomography.

## Discussion

Accurate risk stratification is of great value in formulating individualized treatment plans and evaluating prognoses. Currently, commonly used risk classification systems are based on a patient’s clinical stage, GS, and pretreatment PSA level. However, GS accuracy using random needle core biopsies is not always reliable for the evaluation of PCa. Some patients may also refuse biopsy due to their advanced age or if their cancer was first detected at a late clinical stage. Furthermore, the results of the digital rectal examination depend on the experience and interpretation of the examiner, with non-negligible examiner differences (32). As a non-invasive imaging examination method, PSMA-PET/CT is expected to provide an objective and accurate imaging index for the diagnosis of high-risk PCa.

In this retrospective study of PCa patients, we found that semi-quantitative parameters of primary tumors derived from ^18^F-PSMA-1007 PET/CT, such as SUVmax, TL-PSMAp, and PSMA-TVp, were positively correlated with GS and PSA levels. Furthermore, primary tumor SUVmax, TL-PSMAp, and PSMA-TVp can be used as semi-quantitative imaging biomarkers to identify high-risk PCa. To the best of our knowledge, this is the first retrospective study to evaluate the value of ^18^F-PSMA-1007 PET/CT non-invasive imaging in PCa risk stratification, using primary tumor semi-quantitative parameters SUVmax, TL-PSMAp, and PSMA-TVp.

Recent studies have found that the expression level of PSMA in PCa is positively correlated with tumor stage, GS, and pretreatment PSA level. The increased PSMA expression is associated with a higher degree of malignancy (7, 33, 34). Perner et al. have suggested that PSMA expression is an independent risk factor for PCa recurrence and metastasis (35). Pretreatment PSA levels and GSs are important indicators for PCa risk stratification; therefore, ^18^F-PSMA-1007 PET/CT has unique application potential in evaluating PCa risk classification. Previous studies have also confirmed that the SUVmax of primary tumors obtained from ^68^Ga- and ^18^F-labeled PSMA-PET/CT was significantly associated with PSA levels and GSs (22, 28, 29, 36, 37). Our results are consistent with theirs.

Except for the common parameter SUVmax, the concepts of PSMA whole-body tumor burden with volumetric parameters, whole-body total lesion PSMA, and whole-body PSMA-derived tumor volume were first described by Schmuck et al. (38). In the above study and in other studies, whole-body PSMA-TV and TL-PSMA values showed a significant correlation with PSA levels and GSs in PCa patients with biochemical recurrence (38–42).

Differently, we explored the correlation of the semi-quantitative parameters of the primary prostate tumors with the PSA levels and the GSs. We found that primary tumor semi-quantitative parameters derived from ^18^F-PSMA-1007 PET/CT were positively correlated with PSA levels, and the correlation between TL-PSMAp and PSA was superior to that between SUVmax and PSMA-TVp. This may be because TL-PSMAp can better reflect tumor burden than SUVmax and PSMA-TVp. These semi-quantitative parameters also showed a moderate correlation with GSs; SUVmax, TL-PSMAp, and GS correlated similarly, while PSMA-TVp was more weakly associated with GS. Liu et al. (28) performed ^68^Ga-PSMA-617 PET/CT for 50 newly diagnosed PCa patients with biopsy and measured the SUVmax, PSMA-TV, and TL-PSMA values of the primary lesions. Their results also showed that these ^68^Ga-PSMA-617 PET/CT-derived semi-quantitative parameters positively correlated with pretreatment PSA values and GSs. Distinctions were that TL-PSMAp and PSMA-TVp had a stronger correlation than SUVmax with GS and pretreatment PSA levels. One possible explanation for these findings is that the GS obtained on biopsy may not have represented the true GS of the tumor. The following factors may affect the accuracy of GS obtained by biopsy: the professional level of pathologists, the inaccuracy of urologists in positioning, the percentage of cancer cells in biopsy samples, and the number of biopsy cores (43). In our study, 10.0% (6/60) of patients who did not undergo radical prostatectomies had the highest GS by biopsy, and 36.7% (22/60) of patients underwent radical prostatectomies. Among these 22 patients, the GS of 5 patients changed. Compared with the GS obtained by puncture, the GS of 4 patients increased by 1 score and that of 1 patient decreased by 1 point. For risk stratification, 1 person was elevated from low risk to intermediate risk and the rest of 4 people were unaffected. Finally, the possibility of patient heterogeneity should be considered.

For PCa risk stratification, Hong et al. (21) used the European Association of Urology guidelines on PCa, which are different from our classification standard, to classify low-intermediate- and high-risk patients and used ^18^F-PSMA-1007 PET/CT to identify non-metastatic high-risk PCa. Although the classification criteria (high-risk PCa clinical T stage ≥T2c *vs.* ≥T3a) and enrolled patients differ (non-metastatic *vs*. with or without metastasis), we obtained similar results (area under the curve [AUC]: 0.829 *vs.* 0.828; optimal cut-off value: 9.05 *vs.* 9.61; sensitivity: 90.4% *vs.* 91.03%; and specificity: 65.3% *vs.* 71.43%); SUVmax has a strong ability to identify high-risk PCa. The AUC values for ^18^F-PSMA-1007 PET/CT-derived SUVmax, TL-PSMAp, and PSMA-TVp in our study were similar to the results reported by Liu et al., with TL-PSMAp having the highest AUC in both studies. Good classification criteria for high-risk PCa requires high sensitivity and appropriate specificity to detect most high-risk PCas without leading to overtreatment (44). In comparing ROC sensitivity and specificity values, we found that our SUVmax specificity (71.43%) was better than that reported by Liu et al. (50%), and both studies had high specificity (>85%). Similar to the results of Liu et al., the sensitivities of PSMA-TVp were slightly lower, and the specificities were higher, but the sensitivities and specificities of TL-PSMAp in both studies were satisfactory (28). These results suggest that compared with ^68^Ga-PSMA-617 PET/CT, ^18^F-PSMA-1007 PET/CT shows equally good potential for PCa risk stratification and that TL-PSMAp has the best potential for identifying high-risk patients. This finding was consistent with the previous finding (38, 40). Schmuck et al. indicated that compared with SUVmax, the PSMA-derived volume parameters, TL-PSMAp and PSMA-TVp, could better quantify whole-body tumor burden and facilitate therapeutic monitoring (38). Schmidkonz et al. (40) found that the degree of change in TL-PSMA (87%) was more consistent with the change in PSA level than SUVmax (74%) in their study to evaluate treatment response of PCa treatment using PET/CT parameters. Schmuck et al. obtained similar results (38).

The potential advantages of TL-PSMA can be explained by the fact that SUVmax represents only the highest voxel value within a tumor lesion, namely the maximum metabolic activity, and PSMA-TV represents only the high metabolic volume within the tumor lesion, whereas TL-PSMA combines both metabolic activity and volume to reflect the tumor burden more comprehensively and confirming the previous finding of Im et al. (17).

The present study has some limitations that should not be neglected. Firstly, this was a small retrospective study, and the unbalanced data of LIR patients in this study may affect its statistical power. Secondly, only a small proportion of patients underwent radical prostatectomies with postoperative pathology, and another small proportion of patients may not have a maximal PSA value (PSA values were undiluted at 100 ng/ml), which may explain the lack of a stronger correlation between SUVmax, TL-PSMA, PSMA-TV, and GS/PSA values. Therefore, further validation is required in multicenter prospective studies with larger sample sizes and a greater variety of patient types.

## Conclusions

Based on the significant advantage of ^18^F-PSMA-1007 over ^68^Ga-labeled radiotracers, we investigated its potential for the risk stratification of primary PCas and found that ^18^F-PSMA-1007 PET/CT has a satisfactory application value. The semi-quantitative parameters SUVmax, TL-PSMAp, and PSMA-TVp, were correlated with pretreatment PSA levels and GSs and thus can be used as objective imaging reference indices to determine PCa risk stratification. Furthermore, our findings revealed that TL-PSMAp was most effective in identifying high-risk PCa, providing a better selection basis for prediction of patient prognosis.

## Data availability statement

The raw data supporting the conclusions of this article will be made available by the authors, without undue reservation.

## Ethics statement

The studies involving human participants were reviewed and approved by the Ethics Committee of the General Hospital of Ningxia Medical University, Department of Nuclear Medicine. The patients/participants provided their written informed consent to participate in this study. Written informed consent was obtained from the individual(s) for the publication of any potentially identifiable images or data included in this article.

## Author contributions

Conceptualization and supervision: JL; methodology, data collection, and analysis: SD; identification and PET scan: YLL, JC, and SD; radiopharmaceutical preparation and technical support: PY; PET analysis: YLL, JL, YML, SD, and JC; writing - original draft preparation: SD; writing - review and revision: YML, JL, and SD. All authors contributed to the article and approved the submitted version.
